# Association between dietary supplement use and mortality among US adults with diabetes: a longitudinal cohort study

**DOI:** 10.1186/s12986-023-00753-0

**Published:** 2023-08-11

**Authors:** Rong Hua, Chun Sing Lam, Natural Chu, Aimin Yang, Elaine Chow, Yin Ting Cheung

**Affiliations:** 1 School of Pharmacy, Faculty of Medicine, The Chinese University of Hong Kong, 8th Floor, Lo Kwee-Seong Integrated Biomedical Sciences Building, Area 39, Hong Kong SAR, China; 2 Department of Medicine and Therapeutics, The Chinese University of Hong Kong, Hong Kong SAR, China; 3 Hong Kong Institute of Diabetes and Obesity, The Chinese University of Hong Kong, Hong Kong SAR, China; 4 Phase 1 Clinical Trial Centre, The Chinese University of Hong Kong, Hong Kong SAR, China

**Keywords:** Complementary and alternative medicines, Dietary supplement, Mortality, Diabetes

## Abstract

**Background:**

Despite the popularity of dietary supplements, their effectiveness and safety in patients with diabetes remain controversial. Furthermore, evidence from clinical trials may not be generalizable to real-world settings. This study examined the association between dietary supplement use and mortality outcomes among patients with diabetes based on a nationally representative sample of US adults.

**Methods:**

This study analyzed data from National Health and Nutrition Examination Survey (NHANES) 1999–2018. Supplement users referred to adults with diabetes who reported the use of any dietary supplements in the last 30 days, and with a cumulative duration of ≥ 90 days. Cox proportional hazards models were used to estimate hazard ratios (HRs) and 95% confidence intervals (CIs) for the associations between supplement use and all-cause mortality, and mortality from cardiovascular diseases (CVD), diabetes, and cancer. Subgroup analysis of different supplement classes (vitamins, minerals, botanicals, amino acids, fatty acids, probiotics and glucosamine) were also conducted.

**Results:**

We included 8,122 adults with diabetes (mean age: 59.4 years; 48.7% female), of whom 3,997 (54.0%) reported using supplements regularly. Vitamins (87.3%), minerals (75.3%) and botanicals (51.8%) were the most popular supplements. At a median follow-up of 6.9 years, 2447 all-cause deaths had occurred. Overall supplement use was not associated with risk of all-cause mortality among patients with diabetes (HR = 0.97, 95% CI: 0.87 to 1.08, *P* = 0.56). Subgroup analyses suggested that amino acid use was associated with a lower all-cause mortality (HR = 0.66, 95% CI: 0.46 to 0.96, *P* = 0.028), while the use of fatty acids (HR = 0.62, 95% CI: 0.42 to 0.92, *P* = 0.018) and glucosamine (HR = 0.69, 95% CI: 0.51 to 0.95, *P* = 0.022) supplements were significantly associated with lower CVD mortality.

**Conclusions:**

Our results derived from real-world data suggested that overall supplement use was not associated with any mortality benefit in patients with diabetes. However, there is preliminary evidence that suggests a protective effect of amino acid use on all-cause mortality, and a benefit of fatty acids and glucosamine supplement use on CVD mortality. Future large-scale longitudinal studies are needed to investigate the association between dietary supplement use and other intermediate diabetes-related outcomes, such as glucose control and reducing diabetes-related complications.

**Supplementary Information:**

The online version contains supplementary material available at 10.1186/s12986-023-00753-0.

## Introduction

Among patients with diabetes, it is vital to strictly control hyperglycemia to prevent diabetes-related complications and to reduce mortality from infections, cardiovascular diseases (CVD), stroke, chronic kidney disease, and cancer [1, 2]. In addition to antidiabetic medications, patients with diabetes might also use complementary and alternative medicines (CAMs) to help to manage their diabetes and other chronic health conditions [3, 4]. Among the wide range of CAM products, dietary supplements are popular among patients, as most supplement products have a good safety profile and are easily accessible and affordable [3, 5]. Americans were found to spend nearly $50 to $60 billion on dietary supplements annually [6, 7]. One previous study demonstrated that nearly 54% of US adults with diabetes currently take at least one dietary supplement [8].

Despite the popularity of dietary supplements, their effectiveness and safety in patients with diabetes remain controversial. A few randomized controlled trials have suggested that several vitamins and minerals, such as Vitamin D and zinc, benefit glucose and lipid control [9–11]. However, evidence from clinical trials may not be generalizable to real-world settings in which people often use multiple types of supplements for prolonged periods [5, 12]. Furthermore, previous observational studies have mainly been limited to a single vitamin or mineral supplement [13–16], and there are only a few studies of herbal and other types of supplements [17, 18]. Studies in the general population and of other diseases (such as cancer) have yielded inconsistent results regarding the mortality benefit of supplement use [17–20], and little is known about supplement use in the people with diabetes.

Therefore, to mimic real-world scenarios in which patients often take multiple types of supplements, we used data from a nationally representative sample of US adults to investigate the patterns of dietary supplement use and evaluated their associations with mortality outcomes among patients with diabetes. We also investigated the associations between the use of different classes of supplements (vitamins, minerals, botanicals, amino acids, fatty acids, probiotics, and glucosamine) and mortality risk among patients with diabetes.

## Methods

### Study population

We conducted a prospective cohort study using 10 cycles of survey data (from 1999 to 2000 to 2017–2018) in the US National Health and Nutrition Examination Survey (NHANES). NHANES (https://www.cdc.gov/nchs/nhanes/index.htm) is a continuous, cross-sectional, representative survey of the non-institutionalized US population. NHANES was approved by the the National Center of Health Statistics (NCHS) Ethics Review Board. All NHANES participants provided written informed consent. This study was reported according to the Strengthening the Reporting of Observational Studies in Epidemiology (STROBE) guideline [21].

In the current analysis, diabetes was defined as a self-reported diagnosis of diabetes by a physician or other health professional, the use of any antidiabetic medications, a fasting glucose level ≥ 7.0 mmol/L, or a glycated hemoglobinA_1c_ (HbA_1c_) level ≥ 6.5% [2, 22]. Supplemental Fig. 1 shows the flow chart of participants selection. The sample included 8,716 US adults with diabetes (≥ 20 years old and not pregnant) for whom complete information on mortality outcomes and the use of dietary supplements was available. To capture chronic dietary supplement users, among the 4,591 participants who responded “Yes” to dietary supplement use, we further excluded those who reported using supplements for fewer than 90 days cumulatively (n = 594). Finally, a total of 8,122 NHANES participants were included in this analysis (Table 1).

**Table 1:** Baseline characteristics of patients with diabetes

Characteristics	All patients (n = 8,122)	Supplement Users (n = 3,997)	Supplement Non-users (n = 4,125)	*P* ^*a*^
**Demographics factors**				
Age, years	59.42 ± 0.23	62.31 ± 0.30	56.02 ± 0.29	**< 0.001**
Female (%)	3,927 (48.7)	2,066 (51.4)	1,861 (45.5)	**< 0.001**
Race				**< 0.001**
Mexican American (%)	1,604 (9.0)	629 (6.1)	975 (12.5)	
Non-Hispanic White (%)	2,918 (61.4)	1,719 (69.3)	1,199 (52.2)	
Non-Hispanic Black (%)	2,106 (15.3)	917 (11.7)	1,189 (19.5)	
Others	1,494 (14.2)	732 (12.8)	762 (15.9)	
**Socioeconomic factors**				
Family income to poverty ratio				**< 0.001**
≤ 1.3 (%)	2,640 (23.9)	1,033 (17.3)	1,607 (31.7)	
1.3–3.5 (%)	3,748 (44.0)	1,898 (44.7)	1,850 (43.3)	
> 3.5 (%)	1,734 (32.1)	1,066 (38.0)	668 (25.0)	
Educational level				**< 0.001**
Lower than high school (%)	3,054 (25.7)	1,125 (18.4)	1,929 (34.2)	
High school or equivalent (%)	1,882 (25.8)	961 (25.9)	921 (25.6)	
Above (%)	3,186 (48.6)	1,911 (55.7)	1,275 (40.2)	
**Lifestyle factors**				
BMI status				0.46
< 25 kg/m^2^ (%)	1,173 (12.4)	597 (12.6)	576 (12.2)	
25–30 kg/m^2^ (%)	2,419 (26.9)	1,203 (27.5)	1,216 (26.1)	
≥30 kg/m^2^ (%)	4,530 (60.7)	2,197 (59.8)	2,333 (61.8)	
Smoking status				**< 0.001**
Never smokers (%)	4,031 (49.0)	2,032 (49.1)	1,999 (48.9)	
Former smokers (%)	2,775 (34.2)	1,530 (39.6)	1,245 (27.9)	
Current smokers (%)	1,316 (16.8)	435 (11.3)	881 (23.3)	
Drinking status				0.79
Nondrinker (%)	4,511 (49.0)	2,206 (48.8)	2,305 (49.2)	
Low-to-moderate drinker (%)	3,264 (46.3)	1,634 (46.6)	1,630 (45.8)	
Heavy drinker (%)	347 (4.8)	157 (4.6)	190 (5.0)	
Physically activity (%)	1,661 (23.6)	954 (26.8)	707 (19.8)	**< 0.001**
**Clinical factors**				
Chronic diseases status				
Hypertension (%)	5,876 (69.9)	3,033 (74.4)	2,843 (64.7)	**< 0.001**
Hypercholesterolemia (%)	4,305 (55.4)	2,373 (61.5)	1,932 (48.2)	**< 0.001**
CVD (%)	2,182 (24.9)	1,145 (26.8)	1,037 (22.8)	**< 0.001**
Cancer (%)	1,146 (15.8)	693 (19.3)	453 (11.7)	**< 0.001**
Weak/failing kidney (%)	680 (7.1)	399 (8.3)	281 (5.7)	**< 0.001**
Use of antidiabetic medications (%)	4,791 (58.7)	2,525 (62.0)	2,266 (54.8)	**< 0.001**
Number of antidiabetic medications	Median 4 IQR (2, 7)	Median 4 IQR (2, 7)	Median 3 IQR (1, 6)	**< 0.001**
Type of antidiabetic medications				
Sulfonylureas (%)	2,068 (23.4)	1,061 (23.8)	1,007 (22.8)	0.40
Biguanides (%)	2,995 (38.1)	1,583 (40.9)	1,412 (34.8)	**< 0.001**
Insulin (%)	1,442 (18.0)	753 (18.5)	689 (17.5)	0.41
Alpha-Glucosidase Inhibitors (%)	29 (0.3)	17 (0.4)	12 (0.2)	0.11
Thiazolidinediones (%)	616 (7.9)	318 (8.6)	298 (7.2)	0.070
Meglitinides (%)	70 (0.7)	43 (1.0)	27 (0.5)	**0.018**
DPP-4 Inhibitors (%)	260 (3.4)	163 (4.2)	97 (2.6)	**0.023**
Amylin Analogs (%)	6 (0.1)	4 (0.1)	2 (0.1)	0.64
GLP-1 Receptor Agonists (%)	95 (1.7)	57 (2.1)	38 (1.3)	0.079
SGLT-2 Inhibitors (%)	41 (0.6)	22 (0.8)	19 (0.4)	**0.048**
Others (%)	16 (0.2)	7 (0.1)	9 (0.2)	0.56
HbA_1c_, %	7.26 ± 0.03	7.00 ± 0.03	7.59 ± 0.04	**< 0.001**

BMI: body mass index; CVD: cardiovascular diseases; IQR: interquartile range; DPP-4: Dipeptidyl Peptidase 4; GLP-1: glucagon-like peptide-1; SGLT-2: sodium-glucose cotransporter 2; HbA_1c_: hemoglobinA_1c_

Note: All estimates accounted for survey weights of NHANES. Values are presented as number (weighted percentage), mean ± standard error, or median (IQR)

^a^ Characteristics between supplement users and non-users were compared using the Chi-square test, independent samples t-test, or Wilcoxon rank sum test

### Dietary supplement use

NHANES datasets were collected through in-home personal interviews, physical examinations, and laboratory tests in mobile examination centers. During an in-home interview, the participants were asked if they had used any dietary supplements in the last 30 days. The participants who answered “yes” were further asked to report the duration of use and show the containers to the interviewers so they could record the product information. Based on the product label, the ingredients of each supplement were classified into five categories as stipulated by the NHANES: vitamins, minerals, botanicals, amino acids, or “others” (**Supppemental** Table 1) [23]. Among ingredients that were classified under the “others” category, we also further identified supplement classes that have shown beneficial effects on diabetes in the literature; they included fatty acids [24, 25], probiotics [26, 27], and glucosamine [28].

In this study, we excluded paticipants with supplement use < 90 days (6.0%), and defined supplement users as those who had taken any supplements for ≥ 90 days. Non-users were defined as those who reported no supplement use. Users of specific categories of supplements were defined as those who had taken any supplement containing the corresponding category of ingredients for ≥ 90 days.

### Mortality

Mortality outcomes included all-cause and cause-specific (CVD, cancer, and diabetes) mortalities. All deaths were ascertained through December 31, 2019, by linking to the National Death Index using a probabilistic match method [29]. The International Classification of Diseases 10th Revision (ICD-10) was used to determine the underlying cause of death. CVD mortality was defined as ICD-10 codes I00–I09, I11, I13, I20–I51, and I60–I69. Cancer mortality was defined as ICD-10 codes C00–C97. Diabetes mortality was defined as ICD-10 codes E10–E14.

### Covariates

Covariates included sociodemographic information, lifesltyle factors, history of comorbidities, andiabetic medications use, and laboratory test. Sociodemographic information (age, sex, race, family income, and education level) was collected during in-home interviews. Race was categorized as Mexican American, Non-Hispanic White, Non-Hispanic Black, or other. Family income was classified by family income to poverty ratio (PIR) (≤ 1.3 [low income], 1.3–3.5 [middle income], or > 3.5 [high income]) [30]. PIR was the ratio between a household’s self-reported income and the US Census Bureau’s appropriate poverty threshold for a household. Educational level was categorized as lower than high school, high school or equivalent, or above.

The self-reported lifestyle factors included smoking status, alcohol drinking status, and physical activity. Smoking status were categorizedzed as never smokers (< 100 cigarettes in the entire life), former smokers, or current smokers. Alcohol drinking status were grouped into nondrinkers, low-to-moderate drinkers (< 14 drinks/week for men and < 7 drinks/week for women), or heavy drinkers (≥ 14 drinks/week for men and ≥ 7 drinks/week for women) [31]. Physically activity was defined as engaging in moderate or vigorous physical activity for at least l50 minutes per week [32]. Body weight and height were assessed at a Mobile Examination Center. Body mass index (BMI) was calculated by weight (kg)/height^2^ (m^2^) and classified as normal weight (< 25 kg/m^2^), overweight (25–30 kg/m^2^), or obese (≥ 30 kg/m^2^).

In terms of clinical factors, the physical examinations included blood pressure measurements and a blood draw. Hypertension was defined as systolic blood pressure ≥ 140 mmHg or diastolic blood pressure ≥ 90 mmHg, a self-reported diagnosis of hypertension, or the use of anti-hypertension medications. Other comorbidities, including hypercholesterolemia, CVD, cancer, and weak/failing kidney, were defined by self-reported diagnosis. Antidiabetic medication use was defined as use of insulin or oral glucose-loweirng drugs. Plasma HbA_1c_ was measured by high-performance liquid chromatography methods.

### Statistical analysis

The complex survey design, including sample weights, clustering, and stratification, was accounted for in all analyses according to the NHANES instructions [33]. To obtain an accurate variance estimation, we included all NHANES participants in the analytical dataset, and used the appropriate survey estimation commands and weights to restrict our analysis to the subpopulation of interest [34]. The results are presented as numbers (weighted percentage) for categorical variables and as means ± standard error or median (interquartile range [IQR]) for continuous variables. Differences in baseline characteristics between supplement use status groups were compared by the t-test or Wilcoxon rank sum test for continuous variables and by the Rao-Scott chi-square test for categorical variables.

Cox proportional hazards models were used to estimate hazard ratios (HRs) and 95% confidence intervals (CIs) for the associations between overall supplement use (users versus non-) and risk of all-cause mortality and cause-specific mortality (CVD mortality, cancer mortality, and diabetes mortality). Person-time was calculated from the date of the interview to the date of death or the end of follow-up (December 31, 2019), whichever came first. We checked for violation of assumption of proportional hazards using scaled Schoenfeld residual plots [35]. Four multivariable models were run: Model 1 was adjusted for age, sex, and race. Model 2 was further adjusted for educational level and PIR. Model 3 was further adjusted for smoking status, alcohol intake status, physical activity and BMI status. Model 4 was further adjusted for comorbid conditions (hypertension, hypercholesterolemia, CVD, weak/failing kidney, and cancer), use of antidiabetic medications, and HbA_1c_. Multiple imputation by chained equations were conducted to address covariates with missing values (missing proportion < 15%). 20 imputed datasets were generated.

We conducted two subgroup analyses by supplement categories and use of antidiabetic medications. First, we evaluated the associations of specific supplement categories (vitamins, minerals, botanicals, amino acids, fatty acids, probiotics and glucosamine) with risk of all-cause and cause-specific mortalities. Second, we conducted the subgroup analysis stratified by the use of antidiabetic medication (use or no use) to explore the potential influence of antidiabetic medication use.

Consistent with other NHANES studies on people with diabetes [36, 37], we performed a sensitivity analysis to test the robustness of our results by including only participants who reported a diabetes diagnosis by a physician or other health professional (diagnosed-diabetes), and excluding those who were identified by fasting glucose or HbA_1c_ measurement (undiagnosed-diabetes) [37].

All analyses were conducted using SAS 9.4 (SAS Institute Inc.) and R 4.2.1 (R Foundation, Vienna, Austria). A two-sided α value of 0.05 was considered statistically significant.

## Results

### Comparison of baseline characteristics

We included 8,122 US adults with diabetes (mean age 59.42 ± 0.23 years; 48.7% female) in this analysis. Approximately half (n = 3,997, 54.0%) were dietary supplement users (i.e., those who reported the use of any dietary supplements in the past 30 days for ≥ 90 cumulative days).

Compared with non-users, supplement users were significantly older (62.31 years versus 56.02 years, *P* < 0.001) and more likely to be female (51.4% versus 45.5%; *P* < 0.001) and Non-Hispanic White (69.3% versus 52.2%; *P* < 0.001) (Table 1). The supplement users were more likely to have higher family income (*P* < 0.001) and educational level (*P* < 0.001). Compared with non-users, supplement users tended to adopt a healthier lifestyle, as they were less likely to be current smokers (11.3% versus 23.3%; *P* < 0.001) and more likely to be physically active (26.8% versus 19.8%; *P* < 0.001).

The supplement users were more likely to have hypertension, hypercholesterolemia, CVDs, and cancer (all *P* < 0.001). Supplement users also took more types of antidiabetic medications (*P* < 0.001). Compared with non-users, supplement users were more likely to treated by biguanides (40.9% versus 34.8%; *P* < 0.001), dipeptidyl peptidase 4 inhibitors (4.2% versus 2.6%; *P* = 0.023), and sodium-glucose cotransporter 2 inhibitors (0.8% versus 0.4%; *P* = 0.048) (Table 1). There were no significant differences between supplement users and non-users in use of other commonly prescribed antidiabetic medications. The supplement users had lower HbA_1c_ levels than non-users (7.00% versus 7.59%, *P* < 0.001).

### Patterns of supplement use

**Fig. 1:**
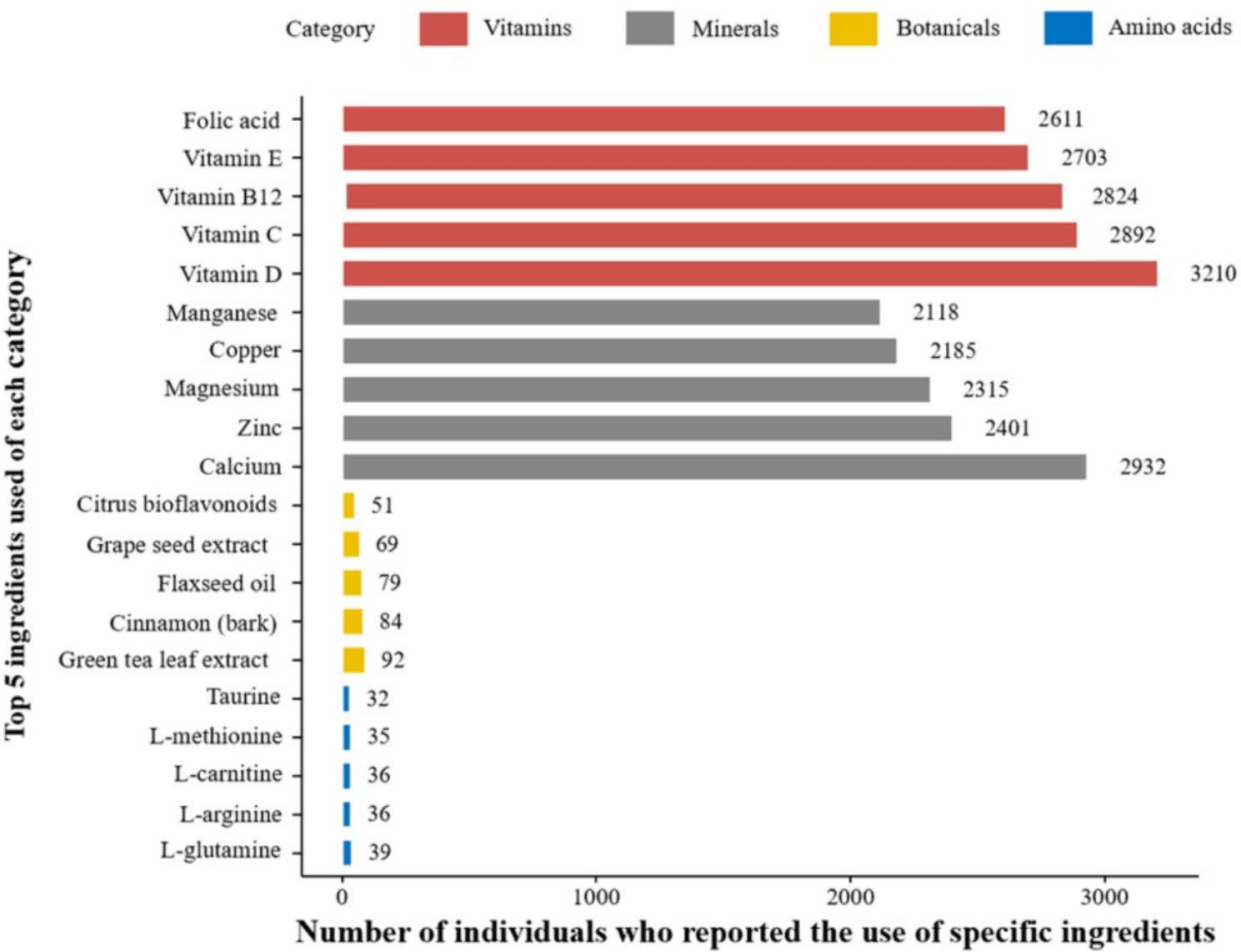
Pattern of supplement use among adults with diabetes in the NHANES cohort

Among the 3,997 supplement users, 1,644 (38.5%) used one supplement, 987 (14.4%) used two supplements, and 1,366 (37.1%) used more than three supplements. Regarding the category of ingredients contained in the supplement, 902 (22.6%) participants used only one category, 622 (15.6%) used two categories, and 2,461 (61.6%) used more than three categories. Specifically, 3,489 (87.3%) participants took vitamins, 3,011 (75.3%) took minerals, 851 (51.8%) took botanicals, and 166 (4.2%) took amino acids. Figure 1 shows the top 5 most used ingredients in each category. Among all ingredients, vitamin D was the most common (n = 3,210). Calcium, green tea leaf extract, L-glutamine were the most commonly used ingredients in the mineral, botanical, and amino acid categories, respectively. In addition, a minority of participants reported using fatty acids (n = 858, 12.3%), probiotics (n = 92, 1.9%), and glucosamine (n = 264, 4.0%). The specific ingredients in different categories of supplements are presented in Supplemental Table 1.

### Associations between overall supplement use and mortality outcomes

During a median follow-up of 6.9 (IQR: 3.5 to 11.4) years, a total of 2,447 (25.8%) participants died, with 836 deaths (8.8%) due to CVD, 402 deaths (4.1%) due to cancer, and 254 deaths (2.9%) due to diabetes. As shown in Table 2, compared with non-users, supplement users were associated with lower risks of all-cause mortality (HR = 0.84, 95% CI: 0.75 to 0.94, *P* = 0.002) and CVD mortality (HR = 0.75, 95% CI: 0.63 to 0.90, *P* = 0.002) after adjusting for age, sex, and race. However, there were no significant associations between supplement use and all-cause mortality after further adjusting for socioeconomic factors (Model 2, HR = 0.94, 95% CI: 0.84 to 1.04, *P* = 0.23), lifestyle factors (Model 3, HR = 0.96, 95% CI: 0.86 to 1.07, *P* = 0.45), and clinical characteristics (Model 4, HR = 0.97, 95% CI: 0.87 to 1.08, *P* = 0.56). Similar non-statistically significant associations were observed for cause-specific mortalities.

**Table 2:** Associations between overall supplement use and mortality outcomes among patients with diabetes

Outcomes	Death among non-users	Death among users	Model 1^a^			Model 2 ^b^			Model 3 ^c^			Model 4 ^d^		
			HR (95% CI)	*P*		HR (95% CI)	*P*		HR (95% CI)	*P*		HR (95% CI)	*P*	
All-cause mortality	1,249 (26.2%)	1,198 (25.5%)	0.84 (0.75, 0.94)	**0.002**		0.94 (0.84, 1.04)	0.23		0.96 (0.86, 1.07)	0.45		0.97 (0.87, 1.08)	0.56	
CVD mortality	438 (9.3%)	398 (8.4%)	0.75 (0.63, 0.90)	**0.002**		0.88 (0.74, 1.06)	0.17		0.91 (0.76, 1.09)	0.31		0.94 (0.78, 1.12)	0.48	
Cancer mortality	189 (4.0%)	213 (4.2%)	0.82 (0.61, 1.10)	0.18		0.96 (0.71, 1.30)	0.79		0.95 (0.71, 1.29)	0.76		0.94 (0.70, 1.27)	0.70	
Diabetes mortality	123 (2.8%)	131 (2.9%)	0.89 (0.61, 1.30)	0.54		1.07 (0.74, 1.55)	0.72		1.11 (0.76, 1.63)	0.59		1.10 (0.75, 1.61)	0.63	

CI: confidence intervals; CVD: cardiovascular diseases; HR: hazard ratio

^a^ Model 1: Adjusted for demographic factors (age, sex, and ethnicity)

^b^ Model 2: Adjusted for demographic factors and socioeconomic factors (educational level and family income to poverty ratio level)

^c^ Model 3: Adjusted for demographic factors, socioeconomic factors and lifestyle factors (smoking status, drinking status, physical activity status, and body mass index status)

^d^ Model 4: Adjusted for demographic factors, socioeconomic factors, lifestyle factors and clinical factors (hypertension, hypercholesterolemia, CVD, weak/failing kidney, cancer, use of antidiabetic medications, and HbA_1c_).

### Subgroup analyses

Table 3 summarizes the associations between different categories of supplements use and risk of all-cause mortality. Only botanical users (*P* = 0.019), amino acid users (*P* = 0.002), and fatty acids users (*P* = 0.007) had significantly lower all-cause mortality after adjusting for demographic and socioeconomic factors. After further adjusting for lifestyle and clinical factors, the protective effect of amino acids remained significant (HR = 0.66, 95% CI: 0.46 to 0.96, *P* = 0.028). The associations for all other supplement categories were not significant in the fully adjusted models. Subgroup analyses were not conducted for probiotics due to the low proportion of deaths among users (n = 15).

**Table 3:** Associations between different categories of supplement use and all-cause mortality among patients with diabetes

Category of supplement use	Total number of users	Death number in users	Model 1 ^a^			Model 2 ^b^			Model 3 ^c^			Model 4 ^d^		
			HR (95%CI)	*P*		HR (95%CI)	*P*		HR (95%CI)	*P*		HR (95%CI)	*P*	
Vitamins	3,489	1,038 (25.6%)	0.83 (0.74, 0.93)	**0.001**		0.93 (0.84, 1.04)	0.20		0.96 (0.85, 1.07)	0.42		0.96 (0.86, 1.08)	0.52	
Minerals	3,011	936 (26.1%)	0.83 (0.73, 0.93)	**0.002**		0.93 (0.83, 1.05)	0.25		0.97 (0.86, 1.09)	0.59		0.98 (0.87, 1.11)	0.73	
Botanicals	851	201 (21.6%)	0.70 (0.57, 0.85)	**0.001**		0.79 (0.66, 0.96)	**0.019**		0.85 (0.70, 1.04)	0.12		0.89 (0.73, 1.09)	0.26	
Amino acids	166	33 (13.2%)	0.49 (0.34, 0.71)	**< 0.001**		0.56 (0.39, 0.81)	**0.002**		0.61 (0.43, 0.87)	**0.007**		0.66 (0.46, 0.96)	**0.028**	
Fatty acids	858	152 (16.2%)	0.61 (0.47, 0.79)	**< 0.001**		0.70 (0.54, 0.90)	**0.007**		0.76 (0.58, 0.99)	**0.045**		0.78 (0.59, 1.03)	0.082	
Glucosamine	264	61 (21.0%)	0.61 (0.43, 0.87)	**0.006**		0.72 (0.51, 1.02)	0.064		0.75 (0.52, 1.09)	0.13		0.78 (0.54, 1.14)	0.20	

CI: confidence intervals; CVD: cardiovascular diseases; HR: hazard ratio

The number and proportion of deaths among non-users are presented in Table 3

Analysis for probiotics is not presented due to the low proportion of deaths among users (n = 15)

^a^ Model 1: Adjusted for demographic factors (age, sex, and ethnicity)

^b^ Model 2: Adjusted for demographic factors and socioeconomic factors (educational level and family income to poverty ratio level)

^c^ Model 3: Adjusted for demographic factors, socioeconomic factors and lifestyle factors (smoking status, drinking status, physical activity status, and body mass index status)

^d^ Model 4: Adjusted for demographic factors, socioeconomic factors, lifestyle factors and clinical factors (hypertension, hypercholesterolemia, CVD, weak/failing kidney, cancer, use of antidiabetic medications, and HbA_1c_).

The associations between the use of different categories of supplements and cause-specific mortalities are summarized in Supplemental Table 2. The use of fatty acids (HR = 0.62, 95% CI: 0.42 to 0.92, *P* = 0.018), as well as glucosamine (HR = 0.54, 95% CI: 0.31 to 0.93, *P* = 0.028), were significantly associated with lower CVD mortality in the fully adjusted model. In addition, botanical use was significantly associated with lower CVD mortality (HR = 0.69, 95% CI: 0.51 to 0.95, *P* = 0.022) than non-users after adjusting for demographic, socioeconomic, and lifestyle factors; a similar trend was observed in the fully adjusted model (HR = 0.75, 95% CI: 0.55 to 1.01, *P* = 0.060), although the association was not statistically significant. The associations for the other supplement categories were not significant in the fully adjusted models.

More than half (n = 4,791, 58.7%) of patients used antidiabetic medications (Supplemental Table 3). Overall supplement use was not associated with any mortality benefits in the fully adjusted model, regardless of the status of antidiabetic medication use.

### Sensitivity analysis

The sensitivity analysis yielded results that were generally consistent with those of the main analyses (Supplemental Table 4). The majority of participants had a confirmed diagnosis of diabetes (n = 6,182, 75.5%). In the fully adjusted model, we observed no significant associations between overall supplement use and all-cause mortality.

## Discussion

In this study, we used longitudinal data from a relatively large nationally representative sample of US adults and found that chronic dietary supplement use was not associated with any mortality benefit among patients with diabetes. Subgroup analyses suggested that amino acid use was associated with a lower all-cause mortality, while the use of fatty acids, glucosamine and botanical supplements were associated with lower CVD mortality. Given the nascent state of the field, our results derived from real-world data may have implications for the development and refinement of recommendations for the use of supplements by patients with diabetes.

The use of dietary supplements was observed in more than half of the study population (54.0%), which is similar to the prevalence estimates reported in another US study [8]. Considering the increasing prevalence of supplement use in the general population [38], this usage rate is expected to rise in the coming years. More evidence on the effectiveness and safety of supplements used in diabetes is needed for clinicians to provide evidence-based recommendations to patients. Our findings show that supplement users on average had a lower HbA_1c_ level than non-supplement users. This may suggest some potential benefits of supplements use on glycemic control. However, whether the lower HbA_1c_ is due to the use of supplements or other coexisting factors such as differences in dietary patterns and lifestyle warrants future research. From a clinical perspective, patients with diabetes typically have a high pill burden and are at risk for polypharmacy. Notably, the supplement users in our study had a higher chronic disease burden and took more types of prescription antidiabetic medications than non-users. The additional use of supplements might further increase their risk of drug interactions or adverse events [39]. Future work should utilize real-world data to evaluate the effects of dietary supplement use on intermediate diabetes-related outcomes, such as glucose control and reducing diabetes-related complications, as well as interactions between commonly used supplements and medications among patients with diabetes.

By considering supplement use as a phenomenon rather than evaluating the effects of individual types of supplements, this study provides novel evidence regarding chronic dietary supplement use and mortality outcomes among patients with diabetes. This approach is further justified by our observation that 55% of the patients used two or more supplements, and 77.4% of the used supplements contained two or more categories of ingredients. Although previous clinical trials have demonstrated the efficacy of certain supplements on diabetes outcomes [11, 13, 14], this study found no association between supplement use and mortality outcomes, which is consistent with observational studies conducted among the general population [16, 17, 19]. For example, using NHANES data, Chen et al. found that overall supplement use was associated with lower mortality in the general population, but the associations were not statistically significant after subsequent adjustments for socioeconomic and lifestyle factors [17]. Similar findings showing no protective effect of supplement use on mortality were also found among Swedish and German populations [16, 19]. An updated meta-analysis also demonstrated that commonly used vitamin and mineral supplements did not confer an all-cause mortality or CVD mortality benefit [40]. These collective findings suggest that the association between supplement use and mortality risk might be confounded by socioeconomic status, lifestyle, and other underlying factors. Consistent with the viewpoints of the American Diabetes Association and the National Center for Complementary and Integrative Health [41, 42], there is still a lack of robust evidence to support or guide the use of dietary supplements in patients with diabetes who do not have underlying deficiencies.

Interestingly, we found a significant association between amino acid use and low risk of all-cause mortality. Compared with extensive studies on the outcomes of vitamin and mineral supplement use, amino acids have been less investigated. Meta-analysis of randomized controlled trials showed that supplementation of amino acids like L-arginine could lower fasting glucose and serum insulin level [43]. The mechanisms underlying this effect might be related to the promotion of adiponectin secretion in adipose tissue, which would increase insulin sensitivity by activating the AMP-activated protein kinase signaling pathway; this might improve glucose uptake and utilization in muscles [44]. Another explanation could be that L-arginine helps to produce nitrogen oxide in the endothelium, thereby improving the capacity of insulin to promote vasodilation [45]. The weighted proportion of amino acid users among diabetic patients was only 2.9%; therefore the possibility of a type I error could not be ruled out, and the results of the multivariable-adjusted models must be cautiously interpreted. Future well-designed studies with larger sample sizes of amino acid users are warranted to verify our findings.

The subgroup analyses also suggested that fatty acids and glucosamine supplement use were associated with lower CVD mortality in this study. In patients with diabetes, the optimal control of cardiovascular risk factors, in addition to glycemic control, may provide greater benefits in reducing morbidity and mortality [41, 46] – this link between diabetics and cardiovascular disorders might underscore the benefits of such supplements on CVD mortality in patients with diabetes. For example, essential fatty acids such as eicosapentaenoic acid (EPA), docosahexaenoic acid (DHA) and alpha linolenic acid were associated with improvements on CVD-related outcomes and glucose control in patients with diabetes [24, 25, 47]. Although glucosamine-containing products are typically used to relieve osteoarthritis and joint pain, some preliminary evidence derived from the UK Biobank showed that regular glucosamine use was associated with lower risks of incident diabetes and CVD events, as well as mortality outcomes [28, 48, 49]. We also observed a trend that botanical supplements might offer potential benefits on mortality (*P* = 0.060). Some plant-based dietary supplements such as grape products and cinnamon have been shown to improve insulin resistance and atherosclerosis, probably by mitigating antioxidative stress and the inflammatory response [50, 51]. Although these preliminary trends are promising, we acknowledge that the effectiveness of botanical supplements requires further study, and potential drug–herb interactions must be considered. Moreover, the usage patterns of botanical dietary supplements might vary among different cultures and ethnicities. While these findings should be interpreted cautiously due to the small sample size, our study provides real-world evidence that fatty acids, glucosamine and botanical supplements might confer CVD mortality benefit among patients with diabetes and warrants further validation prospectively in other cohorts.

The strengths of this study include using validated data from a nationally representative sample of US adults and a longitudinal prospective cohort design to generate real-world evidence. Nevertheless, this study also had some limitations. The use of dietary supplement was evaluated within the last 30 days, which may not represent regular supplement use. To address this limitation, we included only people who had used supplements for ≥ 90 days; in this way, we captured only chronic dietary supplement users. Additionally, supplement use was self-reported, which may have led to misclassification error because of recall bias. However, for most (nearly 80%) NHANES participants [52], the detailed supplement information was derived from the labels on the supplement containers at the time of the interview. The subgroup analysis should be cautiously interpreted due to the small sample sizes of users of botanicals, amino acids, fatty acids, and glucosamine. Furthermore, we were unable to examine the associations between dietary supplement use and mortality from specific CVDs. Although the models were adjusted for several important covariates, other unmeasured and unavailable determinants, such as genetic susceptibility and use of other medications, may confound our results. Finally, the results yielded from the US population might not be generalizable to other countries.

## Conclusion

Based on a nationally representative sample of US adults, we found that more than half of patients with diabetes used dietary supplements. This reflected the popularity of supplement use in diabetic population. Supplement users tend to have more comorbidities and are at a higher risk of medication burden than non-users. Healthcare professionals should proactively communicate with patients on the use of dietary supplements and provide appropriate counselling on the risk of interactions with prescribed medications. Our study found that overall supplement use, specifically vitamin and mineral supplements, was not associated with any mortality benefit in patients with diabetes. However, there is preliminary evidence that suggests a protective effect of amino acid use on all-cause mortality, and a potential benefit of fatty acids, glucosamine or botanical supplement use on CVD mortality. Future large-scale longitudinal studies are needed to investigate the effectiveness of specific supplements in improving intermediate diabetes outcomes, such as glucose control, as well as other long-term complications including cardiovascular, renal and neurological outcomes that can adversely affect the quality of life and health status of patients with diabetes.

This figure presented the top 5 most commonly used ingredients for each category of supplements: vitamins, minerals, botanicals and amino acids. The detailed distributions of ingredients in different categories of supplements are presented in Supplemental Table 1.

### Electronic supplementary material

Below is the link to the electronic supplementary material.

Supplementary Material 1: Supplementary Figures and Tables

## Data Availability

The data used for the current study is publicly available (https://wwwn.cdc.gov/nchs/nhanes/Default.aspx).
