# Supplementary material for: Association between dietary supplement use and mortality among US adults with diabetes: a longitudinal cohort study
Source: Nutr Metab (Lond). 2023 Aug 11;20:33. doi: 10.1186/s12986-023-00753-0 (PMC10416493; doi:10.1186/s12986-023-00753-0)
Supplement: Supplementary file 1 — Supplementary Material 1: Supplementary Figures and Tables [file 12986_2023_753_MOESM1_ESM.docx]

**Supplementary Material**

**Supplemental Figure 1.** Flowchart of participants selection.

**Supplemental Table 1.** Ingredients in different categories of the supplements.

**Supplemental Table 2.** Associations between different categories of supplement use and cause-specific mortality among patients with diabetes.

**Supplemental Table 3.** Associations between overall supplement use and mortality outcomes among patients with diabetes, by antidiabetic medication use status.

**Supplemental Table 4.** Associations between overall supplement use and mortality outcomes among patients with confirmed diagnosis of diabetes.

**Supplemental Figure 1.** Flowchart of participants selection.


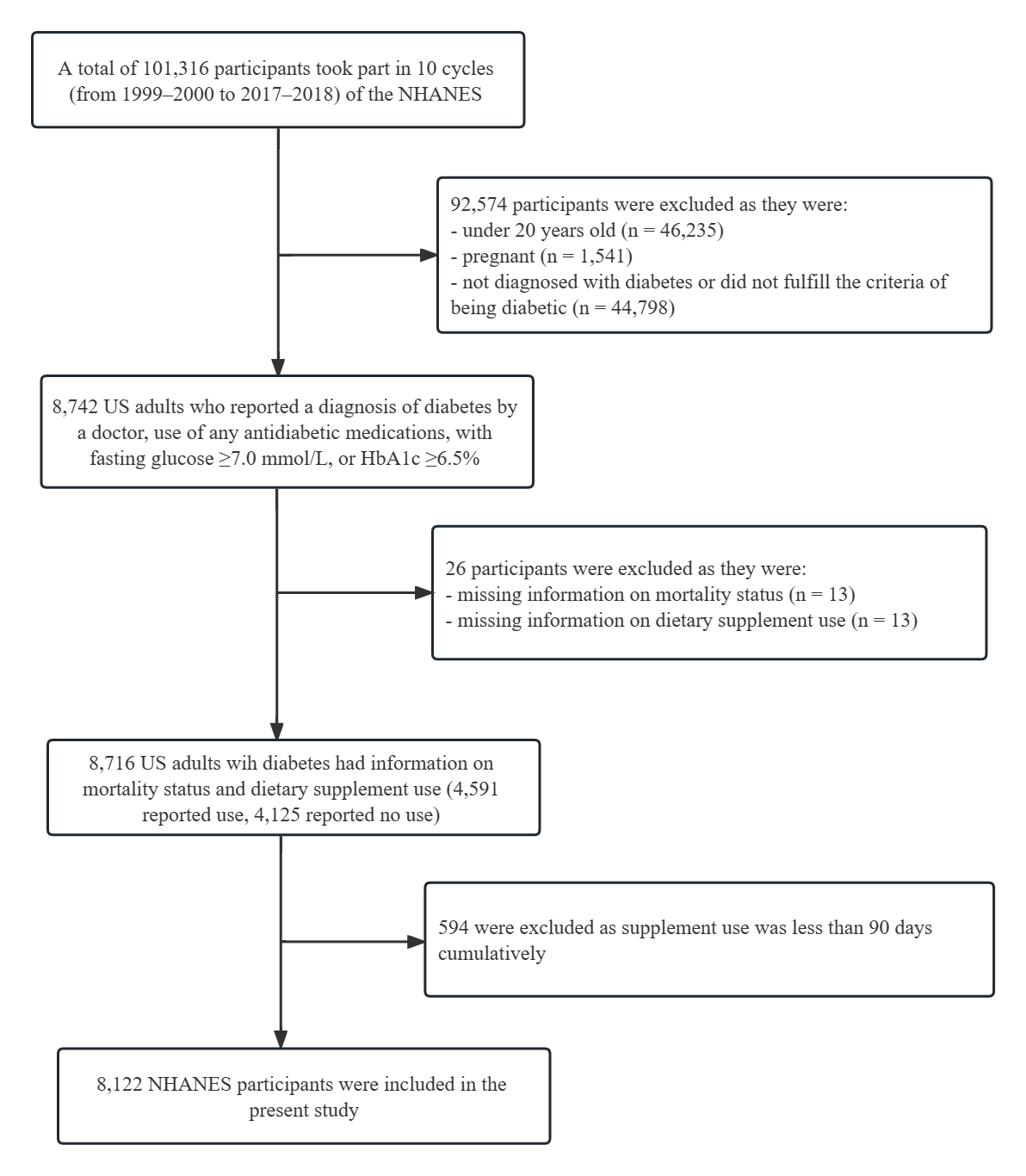


**Supplemental Table 1.** Ingredients in different categories of the supplements

| **Categories** | **Ingredient (*n*)** |
| --- | --- |
| **Vitamins** | Vitamin D (3210), Vitamin C (2892), Vitamin B-12 (2824), Vitamin E (2703), Folic Acid (2611), Vitamin B-6 (2598), Niacin (2518), Vitamin A (2461), Thiamin (2456), Riboflavin (2447), Pantothenic Acid (2444), Biotin (2332), Vitamin K (1898), Choline (191), Choline Bitartrate (103), Mixed Tocopherols (26) |
| **Minerals** | Calcium (2932), Zinc (2401), Magnesium (2315), Copper (2185), Manganese (2118), Chromium (2114), Selenium (2108), Iodine (1859), Molybdenum (1738), Phosphorus (1549), Vanadium (1531), Iron (1458), Boron (1439), Silicon (1392), Nickel (1378), Tin (623), Silica (107), Magnesium Hydroxide (91), Aluminum Hydroxide (83), Calcium Carbonate (74), Magnesium Carbonate (12), Magnesium Oxide (11) |
| **Botanicals** | Green Tea Leaf Extract (92), Cinnamon (Bark) (84), Flaxseed Oil (79), Grape Seed Extract (69), Citrus Bioflavonoids (51), Saw Palmetto Berry Extract (49), Garlic (45), Citrus Bioflavonoid Complex (42), Spirulina (38), Carrot (36), Ginkgo Biloba Leaf (36), Ginkgo Biloba Leaf Extract (35), Spinach (33), Broccoli (32), Kelp (31), Cranberry (30), Turmeric Root (26), Lutein (Flowers) (26), Ginger Root (25), Panax Ginseng Extract (Root) (25), Ginkgo Biloba Extract (Leaf) (25), Boswellia Serrata (Resin) (25), Parsley (24), Cranberry Fruit Extract (24), Bilberry Extract (Fruit) (24), Milk Thistle Seed Extract (24), Pineapple (24), Strawberry (24), Broccoli Powder (23), Garlic (Bulb) (23), Cabbage (23), Orange (23), Blueberry (23), Beet (23), Elderberry Fruit Powder (23), Alfalfa (21), Licorice Root (21), Wheat Grass (21), Brussels Sprouts (21), Grape (21), Turmeric Extract (Root) (21), Green Tea Extract (20), Kale (20), Cauliflower (20), Pomegranate (20), Barley Grass (18), Chlorella (18), Pumpkin Seed Meal (18), Carrot Powder (18), Borage Seed Oil (18), Cherry (18), Carrot (Root) (18), Plum (18), Grape Skin Extract (17), Saw Palmetto Berries (17), Turmeric Root Extract (17), Soy Lecithin (17), Asparagus (17), Bilberry Fruit Powder (17), Alfalfa (Leaf) (16), Pine Bark Extract (16), Tomato (16), Raspberry (16), Acai (16), Evening Primrose Oil (15), Rice Bran (15), Papaya Powder (Fruit) (15), Cauliflower Powder (15), Spinach Powder (14), Green Tea Extract (Leaf) (14), Parsley Leaves (14), Beet (Root) (14), Apple (14), Lemon (14), Brussels Sprout Powder (14), Blueberry Fruit Powder (14), Bilberry (13), Cranberry Concentrate (13), Rose Hips (13), Watercress (13), Odorless Garlic (13), Ginkgo Biloba Powder (Leaf) (13), Rosemary Extract (Leaf) (13), Saw Palmetto (Fruit) (13), Saw Palmetto Extract (Fruit) (13), Citrus Bioflavonoids (Fruit) (13), Apple Fruit Powder (13), Pineapple Fruit Powder (13), Lime (13), Spinach Leaf Powder (13), Kale Powder (13), Kiwi Fruit Powder (13), Odorless Garlic (Bulb) (13), Pomegranate Extract (Fruit) (13), Korean Ginseng Root (12), Pumpkin Seed (12), Ginger Root Powder (12), Green Tea Leaves (12), Ginseng (Root) (12), Flax Seed (12), Papaya (12), Parsley Dehydrate (Stem And Leaves) (12), Echinacea Purpurea (Aerial Parts) (12), Fenugreek Seed (12), Pear (12), Turmeric Extract (Rhizome) (12), Odor Controlled Garlic (Bulb) (12), Olive Leaf Extract (11), Black Currant Powder (11), Pumpkin Seed Oil (11), Lutein (Aerial Parts) (11), Cranberry Concentrate (Fruit) (11), Evening Primrose Oil (Seed) (11), Ginseng (11), Cranberry (Fruit) (11), Borage Oil (11), Orange Fruit Powder (11), Apple Pectin (10), Garlic Powder (10), Shiitake Mushrooms (10), Curcumin (10), Psyllium Husk Fiber (10), Rose Hips (Fruit) (10), Nettle Leaf Powder (10), Ginger Root Extract (10), Grape Seed Extract (Seed) (10), Flax Oil (Seed) (10), Bilberry Extract 4:1 (Fruit) (10), Horsetail Aerial Parts (10), Bilberry Extract (10), Pumpkin Seed Powder (10), Panax Ginseng (Root) (10), Cucumber (10), Grapefruit (10), Tangerine (10), Cranberry Fruit Powder (10), Blackberry (10), Extra Virgin Olive Oil (10), Horsetail Powder (Aerial) (10) |
| **Amino acids** | L-Glutamine (39), L-Arginine (36), L-Carnitine (36), L-Methionine (35), Taurine (32), Glutamic Acid (18), L-Lysine (18), Methionine (18), Glycine (17), L-Cysteine (17), N-Acetyl Cysteine (Nac) (17), N-Acetyl L-Cysteine (17), L-Taurine (16), Arginine (11), L-Isoleucine (10), L-Leucine (10), L-Phenylalanine (10), L-Valine (10), Valine (10), L-Lysine Hcl (10) |
| **Others** |  |
| **Bioflavonoids and Isoflavones** | Hesperidin (35), Bioflavonoids (10) |
| **Electrolyte** | Potassium (1817), Chloride (1457), Sodium (456), Dicalcium Phosphate (11) |
| **Enzyme** | Coenzyme Q10 (178), Lipase (57), Bromelain (52), Cellulase (51), Amylase (49), Papain (46), Protease (37), Lactase (25), Invertase (19), Q-Sorb Coenzyme Q-10 (18), Hemicellulase (10) |
| **Fatty Acids** | Omega-3 Fatty Acids (503), Dha (Docosahexaenoic Acid) (358), Epa (Eicosapentaenoic Acid) (355), Polyunsaturated Fat (332), Fish Oil (310), Saturated Fat (215), Alpha Lipoic Acid (192), Fish Oil Concentrate (184), Other Omega-3 Fatty Acids (134), Monounsaturated Fat (116), Docosahexaenoic Acid (Dha) (111), Eicosapentaenoic Acid (Epa) (106), Alpha-Linolenic Acid (75), Linoleic Acid (74), Natural Fish Oil Concentrate (67), Oleic Acid (62), Omega-3 Epa/Dha (59), Lecithin (50), Epa / Dha (49), Omega-6 Fatty Acids (37), Phospholipids (36), Other Fatty Acids (35), Krill Oil (34), Gla (Gamma-Linolenic Acid) (23), Omega-9 Fatty Acids (21), Phosphatidylserine (16), Omega-9 (Oa) (14), Stearic Acid (13), Cod Liver Oil (12), Palmitic Acid (12), Gamma Linolenic Acid (Gla) (11), Natural Fish Oil (11), Ester Omega Fish Oil (10), Salmon Oil (10) |
| **Glucosamine, Chondroitin and Methylsulfonylmethane (MSM)** | Glucosamine Hydrochloride (214), Chondroitin Sulfate (159), Msm (Methylsulfonylmethane) (138), Chondroitin Sulfate Sodium (46), Glucosamine Sulfate (37), Glucosamine (24), Methylsulfonylmethane (Msm) (13) |
| **Hormone** | Melatonin (56), Dhea (Dehydroepiandrosterone) (13) |
| **Phytochemicals** | Lutein (1263), Rutin (68), Quercetin (54), Betaine Hydrochloride (53), Lutemax 2020 Lutein (33), Resveratrol (29), Hesperidin Complex (24), Chlorophyll (20), Phytosterols (14), Osteo Bi-Flex Joint Shield 5-Loxin Advanced Boswellia Serrata Extract (Resin) (12), Beta Sitosterol (11), Tocotrienol Complex (10), Uniflex (Fruitex-B Calcium Fructoborate) (10), Betaine (10), Policosanol (10) |
| **Probiotics** | Lactobacillus Acidophilus (77), Bifidobacterium Longum (28), Lactobacillus Plantarum (21), Lactobacillus Rhamnosus (20), Bifidobacterium Lactis (18), Bifidobacterium Bifidum (15), Lactobacillus Casei (14), Lactobacillus Salivarius (14), Streptococcus Thermophilus (12), Lactobacillus Acidophilus La-14 (10), Lactobacillus Bulgaricus (10) |
| **Vitamin Precursors** | Beta Carotene-% Of Vitamin A (1971), Lycopene (1153), Zeaxanthin (94), Paba (Para-Aminobenzoic Acid) (92), Astaxanthin (73), Para-Aminobenzoic Acid (Paba) (34), Alpha Carotene (16), Cryptoxanthin (16), Beta Carotene (15), Beta-Cryptoxanthin (13), Mixed Carotenoids Complex (12) |
| **Complexes and Blends** | Calories (1358), Total Fat (818), Calories From Fat (767), Total Carbohydrate (690), Sugars (501), Cholesterol (454), Protein (265), Dietary Fiber (152), Soluble Fiber (63), Collagen (56), Bee Pollen (21), Maltodextrin (15), Liver Powder (12), Royal Jelly (12), Trace Mineral Complex (12) |
| **Other ingredients** | Inositol (309), Hyaluronic Acid (85), Simethicone (76), Brewers Yeast (25), Fos (Fructooligosaccharides) (17), Inulin (15), L-Glutathione (15), Rna (Ribonucleic Acid) (13), Caffeine (12), Hydrolyzed Collagen (12), Joint Fluid (Hyaluronic Acid) (12), Dmae (Dimethylaminoethanol) (11), Gaba (Gamma Aminobutyric Acid) (11) |

*n* refers to the number of individuals who reported the use of the specific ingredients. Only ingredients with a frequency (*n* ≥ 10) are presented.

**Supplemental Table 2. Associations between different categories of supplement use and cause-specific mortality among patients with diabetes**

| **Category of supplement use** | **Death number in users** | **Model 1 ^a^** | |  | **Model 2 ^b^** | |  | **Model 3 ^c^** | |  | **Model 4 ^d^** | |  |
| --- | --- | --- | --- | --- | --- | --- | --- | --- | --- | --- | --- | --- | --- |
|  |  | **HR**  **(95% CI)** | ***P*** |  | **HR**  **(95% CI)** | ***P*** |  | **HR**  **(95% CI)** | ***P*** |  | **HR**  **(95% CI)** | ***P*** |  |
| **Vitamins** |  |  |  |  |  |  |  |  |  |  |  |  |  |
| CVD mortality | 340 (8.4%) | 0.74 (0.62, 0.88) | **0.001** |  | 0.87 (0.72, 1.05) | 0.14 |  | 0.90 (0.74, 1.08) | 0.26 |  | 0.93 (0.77, 1.12) | 0.42 |  |
| Cancer mortality | 180 (4.1%) | 0.78 (0.58, 1.05) | 0.099 |  | 0.92 (0.67, 1.26) | 0.59 |  | 0.91 (0.67, 1.24) | 0.54 |  | 0.90 (0.66, 1.22) | 0.49 |  |
| Diabetes mortality | 118 (3.0%) | 0.89 (0.61, 1.31) | 0.55 |  | 1.08 (0.73, 1.58) | 0.71 |  | 1.12 (0.75, 1.67) | 0.58 |  | 1.11 (0.74, 1.66) | 0.61 |  |
| **Minerals** |  |  |  |  |  |  |  |  |  |  |  |  |  |
| CVD mortality | 318 (8.8%) | 0.77 (0.63, 0.92) | **0.005** |  | 0.89 (0.74, 1.08) | 0.25 |  | 0.93 (0.77, 1.14) | 0.50 |  | 0.96 (0.79, 1.17) | 0.70 |  |
| Cancer mortality | 167 (4.3%) | 0.82 (0.60, 1.11) | 0.20 |  | 0.97 (0.70, 1.36) | 0.87 |  | 0.98 (0.70, 1.36) | 0.89 |  | 0.96 (0.69, 1.33) | 0.82 |  |
| Diabetes mortality | 104 (2.9%) | 0.84 (0.56, 1.26) | 0.41 |  | 1.02 (0.69, 1.51) | 0.91 |  | 1.07 (0.71, 1.60) | 0.75 |  | 1.05 (0.71, 1.57) | 0.80 |  |
| **Botanicals** |  |  |  |  |  |  |  |  |  |  |  |  |  |
| CVD mortality | 65 (6.4%) | 0.55 (0.41, 0.76) | **<0.001** |  | 0.65 (0.48, 0.89) | **0.007** |  | 0.69 (0.51, 0.95) | **0.022** |  | 0.75 (0.55, 1.01) | 0.060 |  |
| Cancer mortality | 37 (4.3%) | 0.84 (0.53, 1.35) | 0.48 |  | 0.96 (0.59, 1.55) | 0.86 |  | 1.03 (0.63, 1.67) | 0.92 |  | 1.02 (0.62, 1.68) | 0.95 |  |
| Diabetes mortality | 22 (2.7%) | 0.85 (0.46, 1.57) | 0.60 |  | 0.97 (0.54, 1.75) | 0.92 |  | 1.05 (0.58, 1.91) | 0.88 |  | 1.18 (0.63, 2.20) | 0.60 |  |
| **Amino acids** |  |  |  |  |  |  |  |  |  |  |  |  |  |
| CVD mortality | 13 (6.4%) | 0.61 (0.30, 1.23) | 0.17 |  | 0.69 (0.36, 1.31) | 0.26 |  | 0.76 (0.41, 1.40) | 0.37 |  | 0.89 (0.47, 1.68) | 0.71 |  |
| Cancer mortality | 7 (2.8%) | / | / |  | / | / |  | / | / |  | / | / |  |
| Diabetes mortality | 2 (0.5%) | / | / |  | / | / |  | / | / |  | / | / |  |
| **Fatty acids** |  |  |  |  |  |  |  |  |  |  |  |  |  |
| CVD mortality | 52 (5.0%) | 0.46 (0.32, 0.66) | **<0.001** |  | 0.56 (0.38, 0.81) | **0.002** |  | 0.60 (0.41, 0.88) | **0.009** |  | 0.62 (0.42, 0.92) | **0.018** |  |
| Cancer mortality | 28 (2.6%) | 0.45 (0.26, 0.79) | **0.005** |  | 0.54 (0.30, 0.98) | **0.044** |  | 0.57 (0.31, 1.04) | 0.065 |  | 0.58 (0.31, 1.06) | 0.075 |  |
| Diabetes mortality | 10 (1.9%) | 0.60 (0.26, 1.39) | 0.23 |  | 0.72 (0.32, 1.63) | 0.43 |  | 0.76 (0.34, 1.72) | 0.51 |  | 0.80 (0.34, 1.86) | 0.60 |  |
| **Glucosamine** |  |  |  |  |  |  |  |  |  |  |  |  |  |
| CVD mortality | 16 (5.0%) | 0.39 (0.22, 0.67) | **0.001** |  | 0.48 (0.28, 0.83) | **0.008** |  | 0.51 (0.29, 0.90) | **0.020** |  | 0.54 (0.31, 0.93) | **0.028** |  |
| Cancer mortality | 11 (3.9%) | 0.61 (0.29, 1.29) | 0.20 |  | 0.73 (0.34, 1.57) | 0.42 |  | 0.73 (0.34, 1.58) | 0.43 |  | 0.72 (0.33, 1.56) | 0.400 |  |
| Diabetes mortality | 5 (1.7%) | / | / |  | / | / |  | / | / |  | / | / |  |

CI: confidence intervals; CVD: cardiovascular diseases; HR: hazard ratio

The number and proportion of deaths among non-users are presented in Table 3.

Analyses of association were not performed if the number of death events <10.

^a^ Model 1: Adjusted for demographic factors (age, sex, and ethnicity).

^b^ Model 2: Adjusted for demographic factors and socioeconomic factors (educational level and family income to poverty ratio level).

^c^ Model 3: Adjusted for demographic factors, socioeconomic factors and lifestyle factors (smoking status, drinking status, physical activity status, and body mass index status).

^d^ Model 4: Adjusted for demographic factors, socioeconomic factors, lifestyle factors and clinical factors (hypertension, hypercholesterolemia, CVD, weak/failing kidney, cancer, use of antidiabetic medications, and HbA_1c_).

**Supplemental Table 3. Associations between overall supplement use and mortality outcomes among patients with diabetes, by antidiabetic medications use status**

| **Antidiabetic medications use** | **Death number in non-users** | **Death number in users** | **Model 1 ^a^** | |  | **Model 2 ^b^** | |  | **Model 3 ^c^** | |  | **Model 4 ^d^** | |  |
| --- | --- | --- | --- | --- | --- | --- | --- | --- | --- | --- | --- | --- | --- | --- |
|  |  |  | **HR (95%CI)** | ***P*** |  | **HR (95%CI)** | ***P*** |  | **HR (95%CI)** | ***P*** |  | **HR (95%CI)** | ***P*** |  |
| **Yes (n=4,791)** |  |  |  |  |  |  |  |  |  |  |  |  |  |  |
| All-cause mortality | 750 (29.0%) | 753 (25.6%) | 0.83 (0.73, 0.95) | **0.007** |  | 0.91 (0.79, 1.04) | 0.18 |  | 0.92 (0.80, 1.06) | 0.27 |  | 0.94 (0.82, 1.08) | 0.42 |  |
| CVD mortality | 288 (11.7%) | 246 (8.6%) | 0.68 (0.55, 0.84) | **0.001** |  | 0.78 (0.62, 0.99) | **0.040** |  | 0.79 (0.63, 0.99) | **0.044** |  | 0.82 (0.66, 1.03) | 0.095 |  |
| Cancer mortality | 99 (3.6%) | 120 (3.7%) | 0.82 (0.54, 1.24) | 0.34 |  | 0.94 (0.62, 1.44) | 0.79 |  | 0.95 (0.62, 1.46) | 0.80 |  | 0.93 (0.61, 1.43) | 0.74 |  |
| Diabetes mortality | 87 (4.1%) | 102 (4.0%) | 0.91 (0.61, 1.36) | 0.65 |  | 1.02 (0.69, 1.51) | 0.91 |  | 1.05 (0.71, 1.57) | 0.80 |  | 1.08 (0.70, 1.67) | 0.73 |  |
| **No (n=3,331)** |  |  |  |  |  |  |  |  |  |  |  |  |  |  |
| All-cause mortality | 499 (22.8%) | 445 (25.4%) | 0.85 (0.72, 0.99) | **0.042** |  | 0.92 (0.78, 1.09) | 0.33 |  | 0.93 (0.79, 1.11) | 0.44 |  | 0.95 (0.80, 1.14) | 0.61 |  |
| CVD mortality | 150 (6.5%) | 152 (8.2%) | 0.90 (0.69, 1.18) | 0.46 |  | 1.04 (0.78, 1.39) | 0.78 |  | 1.03 (0.76, 1.39) | 0.85 |  | 1.05 (0.78, 1.42) | 0.74 |  |
| Cancer mortality | 90 (4.4%) | 93 (5.0%) | 0.84 (0.58, 1.22) | 0.35 |  | 0.92 (0.62, 1.37) | 0.68 |  | 0.90 (0.61, 1.35) | 0.62 |  | 0.92 (0.61, 1.38) | 0.69 |  |
| Diabetes mortality | 36 (1.3%) | 29 (1.2%) | 0.78 (0.38, 1.59) | 0.50 |  | 0.88 (0.39, 1.99) | 0.76 |  | 0.94 (0.40, 2.24) | 0.89 |  | 0.83 (0.35, 1.97) | 0.67 |  |

CI: confidence intervals; CVD: cardiovascular diseases; HR: hazard ratio

^a^ Model 1: Adjusted for demographic factors (age, sex, and ethnicity).

^b^ Model 2: Adjusted for demographic factors and socioeconomic factors (educational level and family income to poverty ratio level).

^c^ Model 3: Adjusted for demographic factors, socioeconomic factors and lifestyle factors (smoking status, drinking status, physical activity status, and body mass index status).

^d^ Model 4: Adjusted for demographic factors, socioeconomic factors, lifestyle factors and clinical factors (hypertension, hypercholesterolemia, CVD, weak/failing kidney, cancer, use of antidiabetic medications, and HbA_1c_).

**Supplemental Table 4. Associations between overall supplement use and mortality outcomes among patients with confirmed diagnosis of diabetes**

| **Mortality Status** | **Death number in non-users** | **Death number in users** | **Model 1 ^a^** | | |  | **Model 2 ^b^** | | |  | **Model 3 ^c^** | |  | **Model 4 ^d^** | |  |
| --- | --- | --- | --- | --- | --- | --- | --- | --- | --- | --- | --- | --- | --- | --- | --- | --- |
|  |  |  | **HR (95%CI)** | | ***P*** |  | **HR (95%CI)** | ***P*** | |  | **HR (95%CI)** | ***P*** |  | **HR (95%CI)** | ***P*** |  |
| All-cause mortality | 1,020  (29.1%) | 975 (26.5%) | 0.82 (0.73, 0.92) | **0.001** | |  | 0.91 (0.81, 1.02) | | 0.12 |  | 0.92 (0.82, 1.04) | 0.19 |  | 0.93 (0.83, 1.05) | 0.25 |  |
| CVD mortality | 375 (11.0%) | 324 (8.9%) | 0.71 (0.58, 0.87) | **0.001** | |  | 0.83 (0.68, 1.02) | | 0.079 |  | 0.85 (0.69, 1.05) | 0.13 |  | 0.88 (0.71, 1.08) | 0.21 |  |
| Cancer mortality | 138 (4.0%) | 162 (4.1%) | 0.82 (0.60, 1.11) | 0.20 | |  | 0.95 (0.69, 1.31) | | 0.75 |  | 0.94 (0.68, 1.30) | 0.71 |  | 0.93 (0.68, 1.27) | 0.65 |  |
| Diabetes mortality | 112 (3.7%) | 120 (3.6%) | 0.85 (0.58, 1.25) | 0.41 | |  | 0.98 (0.66, 1.44) | | 0.91 |  | 0.98 (0.66, 1.47) | 0.94 |  | 1.00 (0.67, 1.49) | 0.99 |  |

CI: confidence intervals; CVD: cardiovascular diseases; HR: hazard ratio

^a^ Model 1: Adjusted for demographic factors (age, sex, and ethnicity).

^b^ Model 2: Adjusted for demographic factors and socioeconomic factors (educational level and family income to poverty ratio level).

^c^ Model 3: Adjusted for demographic factors, socioeconomic factors and lifestyle factors (smoking status, drinking status, physical activity status, and body mass index status).

^d^ Model 4: Adjusted for demographic factors, socioeconomic factors, lifestyle factors and clinical factors (hypertension, hypercholesterolemia, CVD, weak/failing kidney, cancer, use of antidiabetic medications, and HbA_1c_).
